# MSR-RCNN: A Multi-Class Crop Pest Detection Network Based on a Multi-Scale Super-Resolution Feature Enhancement Module

**DOI:** 10.3389/fpls.2022.810546

**Published:** 2022-03-03

**Authors:** Yue Teng, Jie Zhang, Shifeng Dong, Shijian Zheng, Liu Liu

**Affiliations:** ^1^Institute of Intelligent Machines, Hefei Institutes of Physical Science, Chinese Academy of Science, Hefei, China; ^2^Science Island Branch, University of Science and Technology of China, Hefei, China; ^3^Department of Information Engineering, Southwest University of Science and Technology, Mianyang, China; ^4^Department of Computer Science and Engineering, Shanghai JiaoTong University, Shanghai, China

**Keywords:** agricultural pest detection, convolutional neural network, feature enhancement, Soft-IoU, wisdom agriculture

## Abstract

Pest disaster severely reduces crop yield and recognizing them remains a challenging research topic. Existing methods have not fully considered the pest disaster characteristics including object distribution and position requirement, leading to unsatisfactory performance. To address this issue, we propose a robust pest detection network by two customized core designs: multi-scale super-resolution (MSR) feature enhancement module and Soft-IoU (SI) mechanism. The MSR (a plug-and-play module) is employed to improve the detection performance of small-size, multi-scale, and high-similarity pests. It enhances the feature expression ability by using a super-resolution component, a feature fusion mechanism, and a feature weighting mechanism. The SI aims to emphasize the position-based detection requirement by distinguishing the performance of different predictions with the same Intersection over Union (IoU). In addition, to prosper the development of agricultural pest detection, we contribute a large-scale light-trap pest dataset (named LLPD-26), which contains 26-class pests and 18,585 images with high-quality pest detection and classification annotations. Extensive experimental results over multi-class pests demonstrate that our proposed method achieves the best performance by 67.4% of mAP on the LLPD-26 while being 15.0 and 2.7% gain than state-of-the-art pest detection AF-RCNN and HGLA respectively. Ablation studies verify the effectiveness of the proposed components.

## 1. Introduction

The pest disaster is considered as the main reason for crop yield reduction, thus recognizing pests is necessary to guarantee crop yield. Manual pest recognition and location are time-consuming and laborious work. Traditional pest recognition methods prefer to design feature vectors to identify specific pest species, which lacks the generalization ability (Qing et al., 2012; Wang et al., 2012; Yaakob and Jain, 2012; Wen et al., 2015; Deng et al., 2018). Differently, deep learning-based methods using object detection as a ready-to-use approach cause unsatisfied performance due to the enormous gap between pest detection and generic object detection, which could be summarized into the differences in object characters and detection requirements.

The gaps of object characters include small-size, multi-scale, and high-similarly. Small size is the most distinguished property of general object detection. Taking the PASCAL VOC dataset (Everingham et al., 2010) and the LLPD-26 dataset we build as an example, the average size of pests (annotated by bounding boxes) is 1.58% of the general object bounding boxes. Existing methods fail to pay close attention to the small-size pests, which leads to insufficient recognition accuracy. The multi-scale property is another difference between pest detection and general object detection. The object size distribution is wide in pest detection tasks (e.g., the size of Gryllotalpa Orientalis Burmeister is 32 times larger than that of Nilaparvata Lugens Stal in our LLPD-26 dataset). Existing pest detection methods usually use feature fusion of adjacent layers to solve the multi-scale problem, but this fusion method is not sufficient to fully integrate information from different feature layers. The high similarity of interclass is also a crucial challenge (such as Mythimna Separata and Helicoverpa Armigera). Due to the low discrimination ability of high-similarly pests, the performance of the existing methods makes it unsuitable for practical application and remains to be improved.

**Figure 1:**
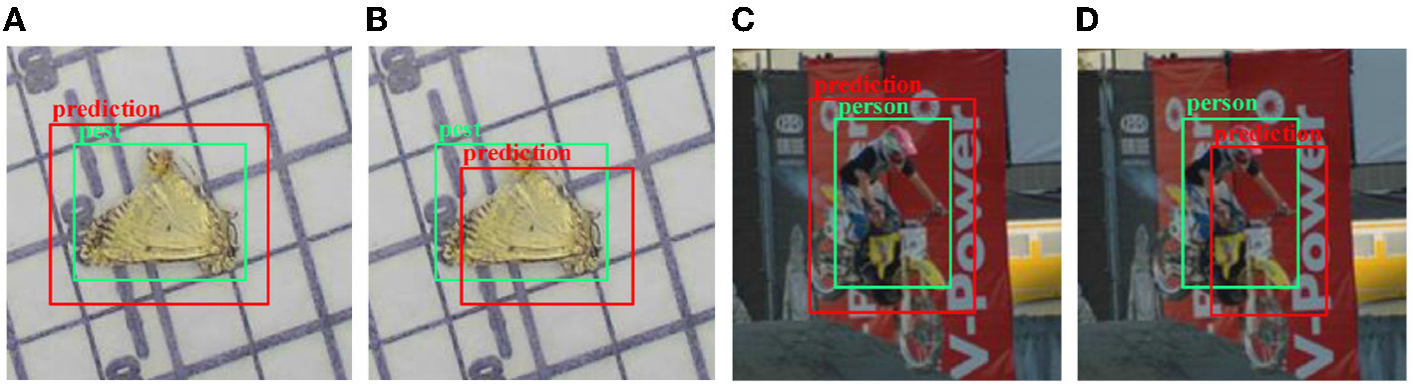
The schematic diagram of different prediction bounding boxes with the same Intersection over Union (IoU). **(A)** The prediction box contains all object pixels. **(B)** The prediction box contains almost all object pixels. **(C)** The prediction box contains pixels of another category (motorbike). **(D)** Most of the pixels in the prediction box are other categories (motorcycles).

Furthermore, position attention is more crucial for pest detection than the high-value Intersection over Union (IoU) compared to general object detection. Different prediction bounding boxes with the same IoU value have diverse performance, as shown in Figure 1. All the predicted bounding boxes (red boxes) in Figure 1 have the same IoU value, but it is clear that the pest detection results are more accurate than the general object detection because there are lesser irrelevant pixels of other categories enclosed (as shown in Figure 1D). The result of Figure 1A is more accurate than the result of Figure 1B because Figure 1A contains all of the pest pixels. Therefore, detection bounding boxes with low IoU hardly cause trouble for pest detection since it excludes other class pixels. Existing methods usually adopt the hard IoU threshold to determine positive and negative samples. By doing so, it could cause some high-quality bounding boxes to be taken as negative samples.

In summary, this study focuses on reducing the gaps between general object detection and pest detection in two dimensions (pest bounding box character and detection target) to improve the performance of pest detection. In pest bounding box dimension: (1) Existing pests detection methods and general object detectors usually utilize FPN (Lin et al., 2017a) to improve the multi-scale feature extraction ability by top-to-down adjacent feature fusion method, but the incomplete fusion limits the performance of detectors. (2) High-similarly objects are recognized using channel attention (Hu et al., 2018) in the general detection field, but the single dimension attention is insufficient for pest detection. (3) The pattern of 5-layer feature maps is employed to detect objects, in which the top layer is used to recognize large-size objects and the down layer is used to recognize small-size objects, but the pest's size is far less than general objects (like dog and cat) resulting in the feature gradually disappearing with the convolution operation. In the pest detection target dimension, pest detections pay more attention to position rather than high-value IoU. Existing methods use a hard IoU threshold to distinguish positive and negative samples resulting in inadequate detection performance. To solve the defect of existing pest detection methods, we propose an MSR-RCNN to improve the detection performance of small-size, multi-scale, and high-similarly pests. The MSR module, the highlight of MSR-RCNN, is a plug-and-play component and can improve the performance of familiar detectors. We first use the super-resolution method to enhance small-size features. Multi-level features are fused at once by feature full fusion mechanism to promote the information transition and high-similarly pests are adequately recognized by feature full weighting mechanism to enhance feature expression ability. In addition, SI is a new design to distinguish different predict bounding boxes with the same IoU value and make networks more suitable for pest detection. Furthermore, to promote the development of pest detection and verify the feasibility of our methods, we construct a large-scale light-trap pest dataset (named LLPD-26) including 18,585 images and 26 classes. Abundant experiments on the LLPD-26 show that our methods can effectively detect multi-class pests and attain start-of-the-art (SOTA) performance.

The main contributions are listed as follows:

- We propose a novel pest detection network (named MSR-RCNN) to solve the defect that existing methods lack the targeted improvement of pest objects in three dimensions: small-size, multi-scale, and high-similarly. The highlight of our MSR-RCNN is the multi-scale super-resolution (MSR) feature enhancement module that can improve the performance of familiar detectors by plug-and-play pattern. The MSR module consists of the super-resolution component, the feature full fusion mechanism, and the feature full weighting mechanism. The three parts focus on improving the performance of small-size, multi-scale, and high-similarly pests.
- Since pest detection focus on the position rather than high-value IoU, we design a SI to differentiate the performance of different prediction result with the same IoU. The SI generates high-quality bounding boxes for network training and employs suitable results to test for pest detection. By using the Soft-IoU, our MSR-RCNN is more fit for pest detection tasks. Meanwhile, the performance of the network is improved without other costs.
- To more accurately monitor and detect multi-class crop pests, we construct a large-scale light-trap pest dataset (named LLPD-26) including 18,585 images and 26 classes. The most-species and largest-number characters of LLPD provide conditions for accurately detecting pests. In addition, adequate experiments on the LLPD-26 verify that our MSR-RCNN outperforms other SOTA methods.

## 2. Related Study

### 2.1. Deep Learning-Based Object Detection

Pest detection is a specific task of general object detection. In recent years, Convolutional Neural Network (CNN) is widely applied in the object detection fields. The deep learning-based object detection networks divide into one-stage networks and two-stage networks. As one of the most famous networks in the one-stage, Redmon et al. (2016) utilized the whole image as the input and directly obtained the prediction result using 24 convolution layers and 2 full connection layers. Subsequently, some enhanced versions of YOLO were proposed one after another (Redmon and Farhadi, 2017, 2018; Bochkovskiy et al., 2020). Lin designed Retinanet to solve the problem of positive and negative sample imbalance with the Focal Loss, thus improving the detection accuracy (Lin et al., 2017b). The FCOS avoided the anchor mechanism with the pattern of point regression resulting in reducing the number of hyperparameters. Meanwhile, low-quality predictions were filtered out through the proposed Center-ness branch (Tian et al., 2019). Two-stage networks require the selective search (Uijlings et al., 2013) or region proposal network (RPN) to generate region proposal first, and then the R-CNN network (Girshick et al., 2014) is used to refine the proposal box (Girshick, 2015). Faster R-CNN (Ren et al., 2017) proposed RPN based on the Fast R-CNN and established the baseline of the two-stage detector. Pang et al. designed the Cascade R-CNN network to continuously optimize the detection results by gradually increasing the IoU threshold (Cai and Vasconcelos, 2018). Libra R-CNN used concat to merge feature layers, but the essence of the feature fusion method was reducing the video memory for the non-local mechanism (Pang et al., 2019). FPN (Lin et al., 2017a) and PANet (Liu et al., 2018) used feature fusion of adjacent layers to solve the multi-scale problem, but the incomplete fusion method did not meet the requirement of pest detection. TridentNet used dilated convolution (Yu and Koltun, 2015) to improve the capability of multi-scale feature extraction (Li et al., 2019). The ThunderNet used Context Enhancement Module (CEM) module to integrate multi-scale information and adopted the Spatial Attention Module (SAM) to enhance feature representation (Qin et al., 2019). OHEM (Shrivastava et al., 2016) and Snip/Sniper (Singh and Davis, 2018; Singh et al., 2018) improved the performance of the network by using selective backpropagation. We use the two-stage framework as the baseline because the two-stage methods are usually more accurate than the one-stage methods, especially for small-size object detection.

### 2.2. Pest Detection Method Based on CNN

Due to the rapid development of CNN-based object detection, many researchers transplant deep learning-based methods to agricultural applications (Kamilaris and Prenafeta-Boldú, 2018; Dhaka et al., 2021; Hasan et al., 2021). In the pest recognition and detection field, Liu et al. (2016) used a global contrast region-based approach to construct a rice insect classification dataset named Pest_ID and used a CNN to identify the insects. Wang et al. (2017) applied LeNet and AlexNet to classify pest images. Thenmozhi and Reddy (2019) used transfer learning to explore the results of AlexNet, ResNet, LeNet, and VGG on three pest datasets. Yue et al. (2018) proposed a super-resolution method based on deep learning to solve the difficulty of insect recognition. Ayan et al. (2020) combined different CNN networks into a unified pest identification network and automatically selected the combination weight to carry out pest identification *via* the genetic algorithm. Shen et al. (2018) proposed an improved Faster R-CNN network with the inception structure to identify common grain pests. Liu et al. (2019) designed a detection network combining Faster R-CNN and channel-spatial to detect the light-trap pests. Jiao et al. (2020) proposed an anchor-free network (AF-RCNN) to identify and locate pests of 24 types. Liu et al. (2020) used global and local activation features to detect the 16-class pest dataset. The above methods ignore the gaps between object detection and pest detection and use insufficient improvement for pest detection, which led to an unsatisfied performance. Therefore, we design an MSR-RCNN to improve the performance of pest detection.

## 3. Materials and Methods

### 3.1. Data Collection

We use the light-trap device to automatically collect the pest images in different periods. The data collection devices are from the Intelligent Machines Institute, Chinese Academy of Sciences, and distributed in the field environment of Anhui Province. The dataset includes 18,585 JPEG images with the resolution of 2,592 × 1,944 and is annotated by agricultural experts. Each pest object corresponds to a unique category and bounding box coordinate, and each image has multiple pests. To ensure effectiveness, we divide the data into 14,868 images of the train set and 3,717 images of the test set.

### 3.2. MSR-RCNN Pest Detection Network

**Figure 2:**
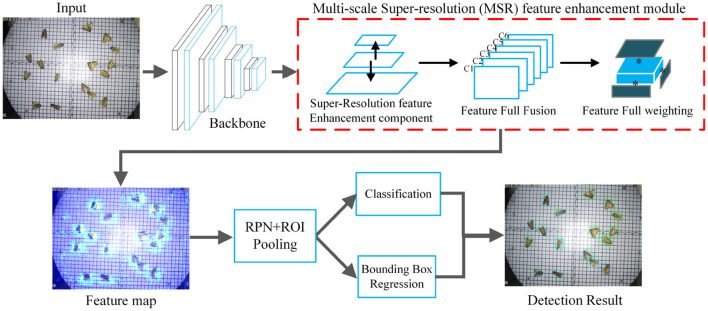
The overall framework of the MSR-RCNN.

To accurately detect 26-class pests, we design an MSR-RCNN network including a backbone network (ResNet50), MSR feature enhancement module, RPN, and bounding box regression and classification networks (RCNN). We use ResNet50 (He et al., 2016) as the backbone network to extract image features. The MSR feature enhancement module is utilized to improve the feature expression ability of the backbone in three dimensions: small-size, multi-scale, and high-similarly. With the MSR module, enhanced features are obtained for pest detection. The RPN (Ren et al., 2017) is used to obtain the region of interest (ROI) and the ROI Align (He et al., 2017) is employed to resize the ROI to the unitive size. Classification branch and bounding box regression are applied to obtain the final detection results, as shown in Figure 2.

### 3.3. MSR Feature Enhancement Module

Since small-size, multi-scale, and high-similarly pest characters of pests, we design the MSR feature enhancement module to improve the detection performance using a super-resolution component, a feature full fusion mechanism, and a feature full weighting mechanism. The super-resolution component from the MSR module obtains the six-layer feature map for the recognition of small size objects. Then, the full feature fusion mechanism integrates all features at once for the recognition of multi-scale objects. Since high-similarly pests in the LLPD-26 dataset are difficult to identify, we design the feature full weighting mechanism in the MSR module to enhance the fine-grained expression ability. The red part of Figure 2 shows the overall framework of the MSR we devised.

#### 3.3.1. Super-Resolution Feature Enhancement Component

Feature pyramid network (FPN) (Lin et al., 2017a) uses 5 layer feature maps to recognize objects, in which the top-level features include semantic information to detect large-size objects and the low-level features include texture information to detect small-size objects. However, the small-size pest features gradually disappear in the process of convolution operation resulting in misleading information transfer in the top-to-down feature fusion. Inspired by zooming in to identify pests in the manual annotation process, we design the super-resolution feature enhancement component to improve small-size feature extraction ability by using deconvolution to obtain fine-grained pest features.

**Figure 3:**
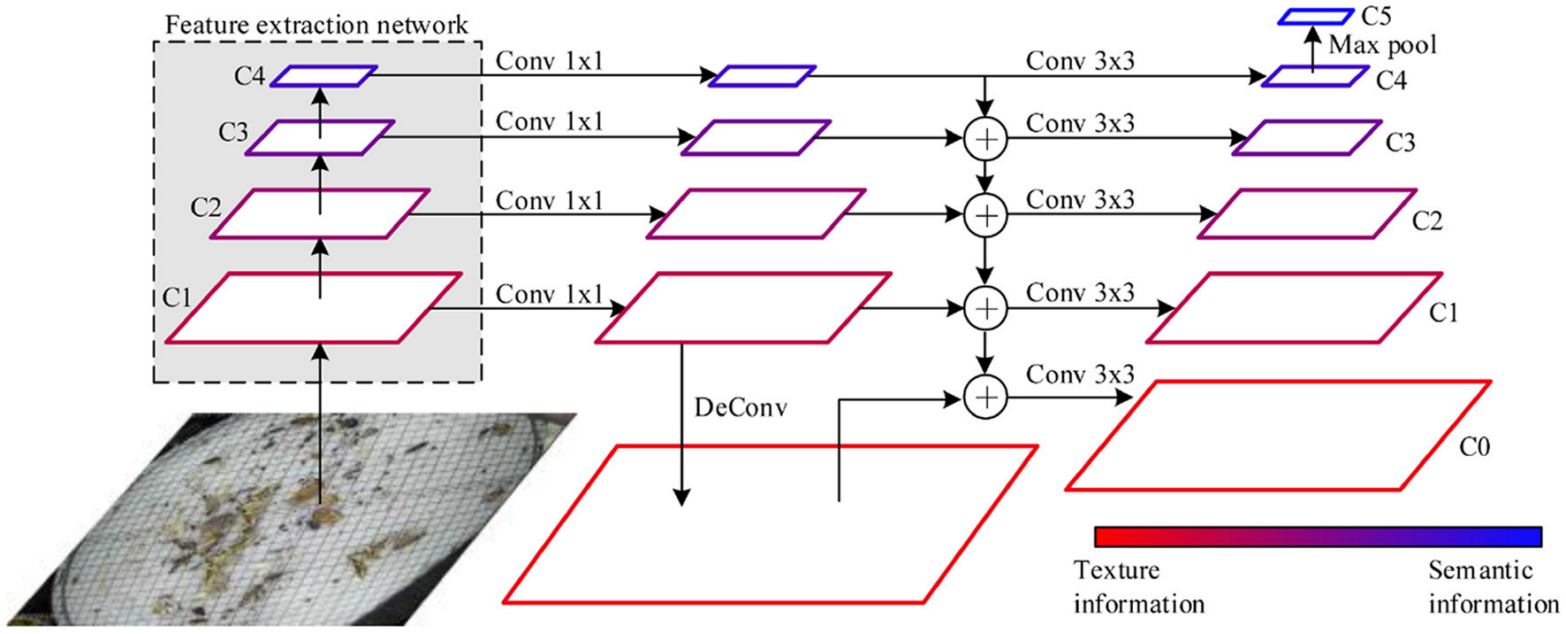
The super-resolution feature enhancement component.

To ensure the full utilization of features, we select the feature maps after each Resnet50 block (a total of 4) as the input of the super-resolution component. We use 1 x 1 convolution kernels for each layer feature to change the number of channels to 256. Duo to the size of pest objects is small, we deconvolve the feature map after the first block of the Resnet50 network to enhance texture information, which refers to the way people zoom in on images for small-size object recognition. In this way, we have 5-layer feature maps, four layers from the feature extraction network, and one layer from deconvolution operation. We use the bilinear interpolation method to add the upper layer features and apply the lower layer features to carry out adjacent layer feature fusion. The 3 x 3 convolution kernel is utilized to enhance the feature representation capability. Max pooling operation is carried out for top layer feature to enhance semantic information. After the above process, we have 6-layer feature maps, in which the top layer feature obtained by max-pooling has sufficient semantic information, and the bottom layer feature obtained by deconvolution has rich texture information. Figure 3 shows the super-resolution feature enhancement component designed in this study.

#### 3.3.2. Feature Full Fusion

**Figure 4:**
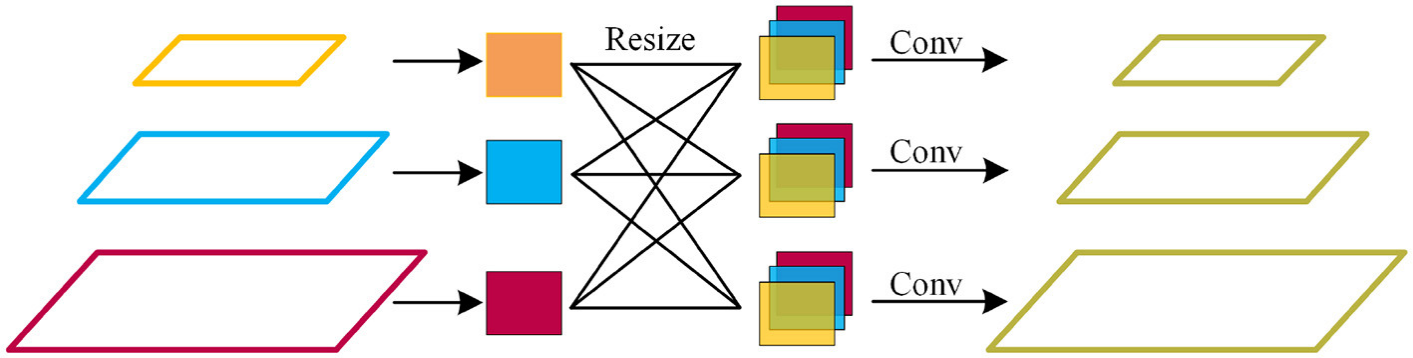
The feature full fusion mechanism.

The feature full fusion mechanism is used to improve the performance of multi-scale pest detection. By fusing the information of different feature layers, the defects are avoided in existing methods, which only combine adjacent layers or use a single feature layer to detect pests (Jiao et al., 2020; Liu et al., 2020). The inspiration for our design comes from the process of people looking at images. People often think of an image as a 2D image because the human eye treats multiple channels (usually RGB, 3-channel) at once. Similarly, the feature full fusion mechanism combines the 6-layer features from the super-resolution component at once. We fuse 6-layer feature maps into five layers to improve network efficiency. Specifically, for each of the 6-layer feature maps, we use the bilinear interpolation method to resize them to five sizes, in which the resolutions are 200 × 272, 100 × 136, 50 × 68, 25 × 34, and 13 × 17, respectively. We stack features of the same size and use a 1 × 1 convolution to unify channels to 256. The stacked feature maps are added to the C1~C5 feature maps of the original feature maps. It is important to note that our feature full fusion is substantially different from the full connection layer, although it is very similar. This is because our feature full fusion module preserves the translation invariance of the pixels. This also leaves enough information for the next feature full weighting module. Figure 4 shows the feature full fusion mechanism.

#### 3.3.3. Feature Full Weighting

Due to the high-similarly pests in the LLPD-26 (e.g., *Cnaphalocrocis medinalis and Pyrausta nubilalis, Mamestra brassicae Linnaeus and Scotogramma trifolii Rottemberg*), fine-grained identification is required to improve the performance of detection. We design the feature full weighting for feature reinforcement learning. This could optimize the detection performance of similar pests from two dimensions (depth and location). For the feature map (*W*, *H*, and *C*) of each layer, our weighting method weights channel *C* and points (*x, y*) in the feature map, where *W* is the width, *H* is the height, and *C* is the channel number of the feature map. We use Formula (1) to describe our weighting method.


(1) $$\begin{array}{r} W\left(X\right)=\alpha \pi_{L}\left(X\right)g\left(X\right)+\left(1-\alpha \right)\pi_{C}\left(X\right)X \end{array}$$

Where π_*L*_(·) represents the local weighting function, π_*C*_(·) represents the channel weighting function, *X* represents the feature map, *W*(*X*) represents the weighted feature map, and α is the scale factor. Formula (2) and Formula (3) give the specific forms of π_*L*_(·) and π_*C*_(·), respectively.


(2) $$\begin{array}{r} \pi_{L}\left(x_{i}\right)=\underset{\forall j\in X}{\sum }\theta_{L}{\left(x_{j}\right)}^{\text{T}}\phi_{L}\left(x_{i}\right) \end{array}$$

(3) $$\begin{array}{r} \pi_{C}\left(X\right)=ReLu\left(\theta_{C}\left(avg\left(X\right)\right)\right)+ReLu\left(\phi_{C}\left(max\left(X\right)\right)\right) \end{array}$$

**Figure 5:**
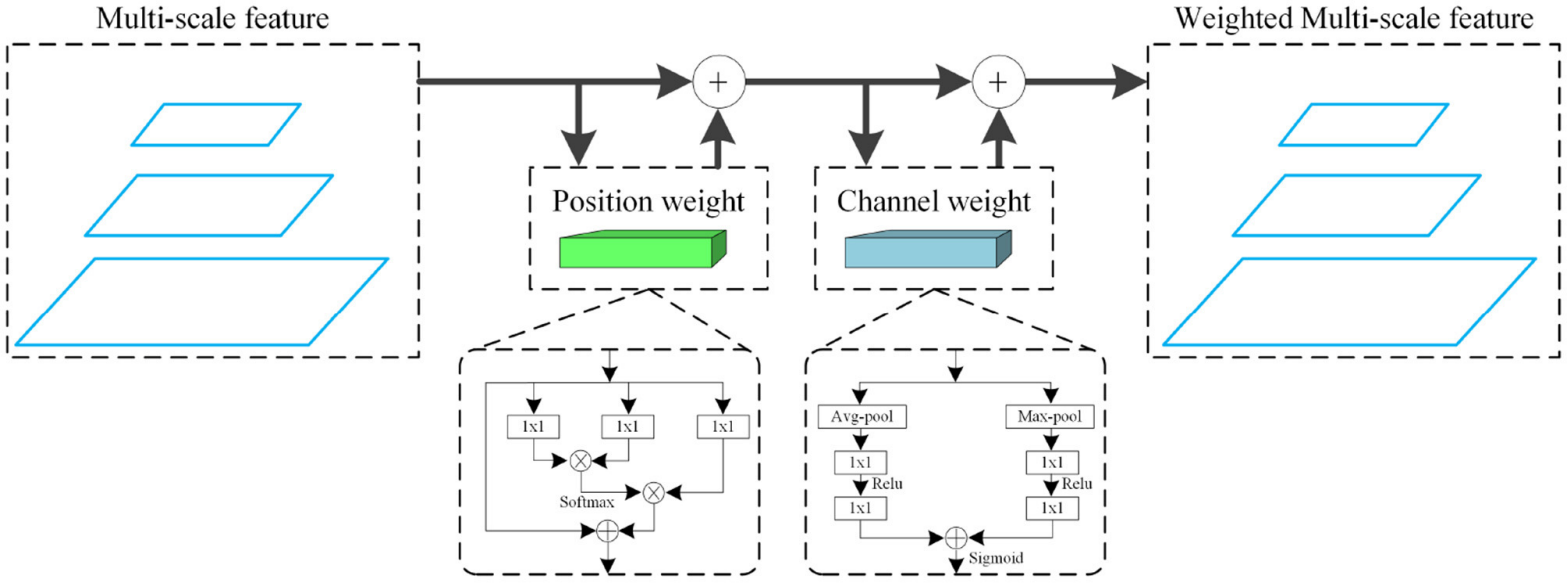
The feature full weighting mechanism.

Among them, *x*_*j*_ represents the point on the feature map excluding the point *X*_*i*_, θ(·) and ϕ(·) represent the learnable function for feature *X*, *avg*(·) and *max*(·) represent global average pooling and global maximum pooling, respectively. To guarantee the end-to-end pattern, we use a convolution operation to carry out the feature full weighting, as shown in Figure 5.

### 3.4. Soft-IoU

In general object detection (such as PASCAL VOC), *IoU*_50_ is used as the threshold to determine positive and negative samples. However, for pest detection, different bounding boxes with the same IoU value have different performances. Therefore, we design a SI with the position suppression method to optimize the training and test processes. Specifically, the calculation method of SI is shown in Formula (4):


(4) $$\begin{array}{r} SI\left(A,B\right)=\beta \cdot \left\lceil 1-\frac{E\left(A_{center},B_{center}\right)}{max\left(A_{diagonal},B_{diagonal}\right)}\right\rceil \cdot \frac{A\cap B}{A\cup B} \end{array}$$

Where *E*(·) represents the Euclidean distance, *A*_*center*_ and *B*_*center*_ represent the center point of bounding box A and B, respectively, *A*_*diagonal*_ and *B*_*diagonal*_ represent the diagonal distance of bounding box A and B, respectively, *Max*(·) represents the maximum function, and β is the scaling factor. To ensure the stability, we adjust the IoU no more than 0.1 times the original IoU. Due to the high-quality positive samples contributing to training the network finely, β is selected as 0.9. In the test phase, β = 1.1 because we expect the bounding box as shown in Figure 1A to output the results as a positive sample.

## 4. Experiments

### 4.1. Experiment Settings

We use the backpropagation and Stochastic Gradient Descent (SGD) to train our MSR-RCNN (LeCun et al., 1989). For the training of MSR-RCNN, each SGD mini-batch is constructed from a single pest image that contains 256 samples. Negative samples and positive samples are randomly selected in a ratio of 1:1 in each mini-batch. Gaussian distribution with a mean of 0 and a SD of 0.01 is used to initialize the parameters of the classification regression layer. In each SGD iteration, we use RPN to generate 1,000 potential regions to be sent to R-CNN for learning. We train a total of 12 epochs with a momentum of 0.9, among which the first 8 epochs have a learning rate of 0.0025, and the last 4 epochs are 0.00025. Our experiment is deployed on a Dell 750 server with NVIDIA Titan RTX GPU (24G memory) using the Mmdetection2.0.0 (Chen et al., 2019) framework and Python 3.8. Unless otherwise stated, all comparison models in this study use the default parameters. Since the SmoothL1 Loss function is differentiable at zero, we use it to train the R-CNN network for more stable performance. Because the L1 Loss is a non-differentiable function at zero, we apply it in RPN network training to improve the robustness.

### 4.2. Experiment Results

#### 4.2.1. Performance on Our LLPD-26

We compare the performance of our method with Faster R-CNN (Ren et al., 2017), Cascade R-CNN (Cai and Vasconcelos, 2018), Libra R-CNN (Pang et al., 2019), FCOS (Tian et al., 2019), Retinanet (Lin et al., 2017b), AF-RCNN (Jiao et al., 2020), and HGLA (Liu et al., 2020), as shown in Table 1. Among them, AF-RCNN and HGLA are the existing deep learning-based pest detection methods, MSR represents the MSR feature enhancement module proposed by us, SI represents the SI, *AP*_50_ represents the Average Precision (AP) with the IoU threshold of 50%, AP represents the mean AP with the IoU threshold at 50, 75, and 95%. The FPN (Lin et al., 2017a) is used in all comparison methods. Our MSR module is slightly inferior to Libra R-CNN in *AP*_75_ performance due to the high-quality training box provided by the balanced sampling approach of Libra R-CNN. In addition, since pest detection is more focused on point location performance than bounding box IoU performance, *AP*_50_ is more valuable than *AP*_75_. With the SI training method, the MSR-RCNN outperforms other methods.

**Table 1:** The overall performance comparison.

**Method**	**MSR**	**SIoU**	***AP***	***AP*_50_**	***AP*_75_**	***mRecall***
*General object detection*						
Faster R-CNN (Ren et al., 2017)			35.4	62.3	37.7	50.5
Cascade R-CNN (Cai and Vasconcelos, 2018)			36.0	62.6	38.5	50.2
Libra R-CNN (Pang et al., 2019)			37.4	65.2	40.2	52.8
FCOS (Tian et al., 2019)			33.3	57.4	36.2	**55.2**
RetinaNet (Lin et al., 2017b)			27.9	48.8	29.4	53.1
*Pest detection*						
AF-RCNN (Jiao et al., 2020)			33.1	58.6	34.6	48.8
HGLA (Liu et al., 2020)			37.0	65.6	38.3	52.0
*Ours*						
MSR-RCNN	√		38.0	66.9	40.0	52.4
MSR-RCNN	√	√	**38.4**	**67.4**	**40.6**	52.0

To compare the performance of the proposed method in detail, the *AP*_50_ results of each category are given in Table 2. We emphasize the best results for each class with bold to show the best performance. It can be found that our network outperforms other methods.

**Table 2:** Compare results by category on our LLPD-26 dataset using *AP*_50_.

**Class number**	**General object detection**				**Pest detection**		**Ours**	
	**Faster R-CNN**	**Cascade R-CNN**	**Libra R-CNN**	**RetinaNet**	**AF-RCNN**	**HGLA**	**MSR**	**MSR+SI**
1	16.1	19.2	21.7	4.5	12.8	20.1	**21.1**	20.4
2	58.7	58.9	63.5	54.4	58.7	63.3	66.1	**67.2**
3	70.2	67.9	70.1	60.9	65.5	71.7	72.8	**74.0**
4	69.6	69.4	70.9	58.0	66.0	72.8	72.3	**72.8**
5	84.9	85.2	85.0	80.7	83.5	86.1	**86.2**	85.8
6	72.1	71.1	74.4	66.0	70.4	76.2	**76.4**	77.4
7	72.5	71.9	73.4	62.4	70.9	74.0	**74.9**	74.5
8	62.0	60.6	66.7	57.5	59.4	66.1	65.5	**70.5**
9	47.5	47.5	50.9	43.0	47.3	51.9	**53.6**	53.5
10	70.9	70.5	74.2	59.6	68.5	74.2	77.2	**78.8**
11	79.3	78.2	80.3	73.2	76.1	81.6	81.0	**81.7**
12	27.7	26.9	26.7	0.10	25.5	**32.7**	29.5	32.3
13	55.3	58.3	54.6	41.5	53.4	55.4	56.8	**59.7**
14	66.7	64.5	66.4	57.3	62.0	67.4	67.5	**69.9**
15	39.8	45.3	47.3	8.10	33.1	45.2	48.0	**51.3**
16	40.2	45.2	51.7	7.50	33.0	50.7	49.6	**51.5**
17	57.9	65.1	66.8	15.0	55.4	70.8	**70.9**	70.6
18	56.1	58.7	60.5	35.9	55.0	58.0	**64.9**	63.3
19	56.6	58.4	64.9	54.3	57.7	61.9	65.1	**69.4**
20	83.0	82.7	82.1	78.1	80.6	83.7	83.3	**83.8**
21	89.5	89.5	89.5	86.9	87.5	90.0	90.0	**90.1**
22	93.1	92.4	94.4	93.8	91.7	94.4	**94.6**	94.4
23	59.9	51.7	63.2	54.1	54.1	61.2	**66.0**	63.9
24	72.8	73.3	74.9	64.1	71.4	74.8	74.0	**75.2**
25	53.3	49.7	54.8	1.20	14.8	50.0	**59.2**	56.4
26	64.8	70.4	65.0	49.6	68.2	70.2	**73.0**	63.1
Mean	62.3	62.8	65.2	48.8	58.6	65.6	66.9	**67.4**

*The parts in bold represent the best performance*.

#### 4.2.2. Ablation Experiments

##### 4.2.2.1. Category Performance Improvement Comparison

**Figure 6:**
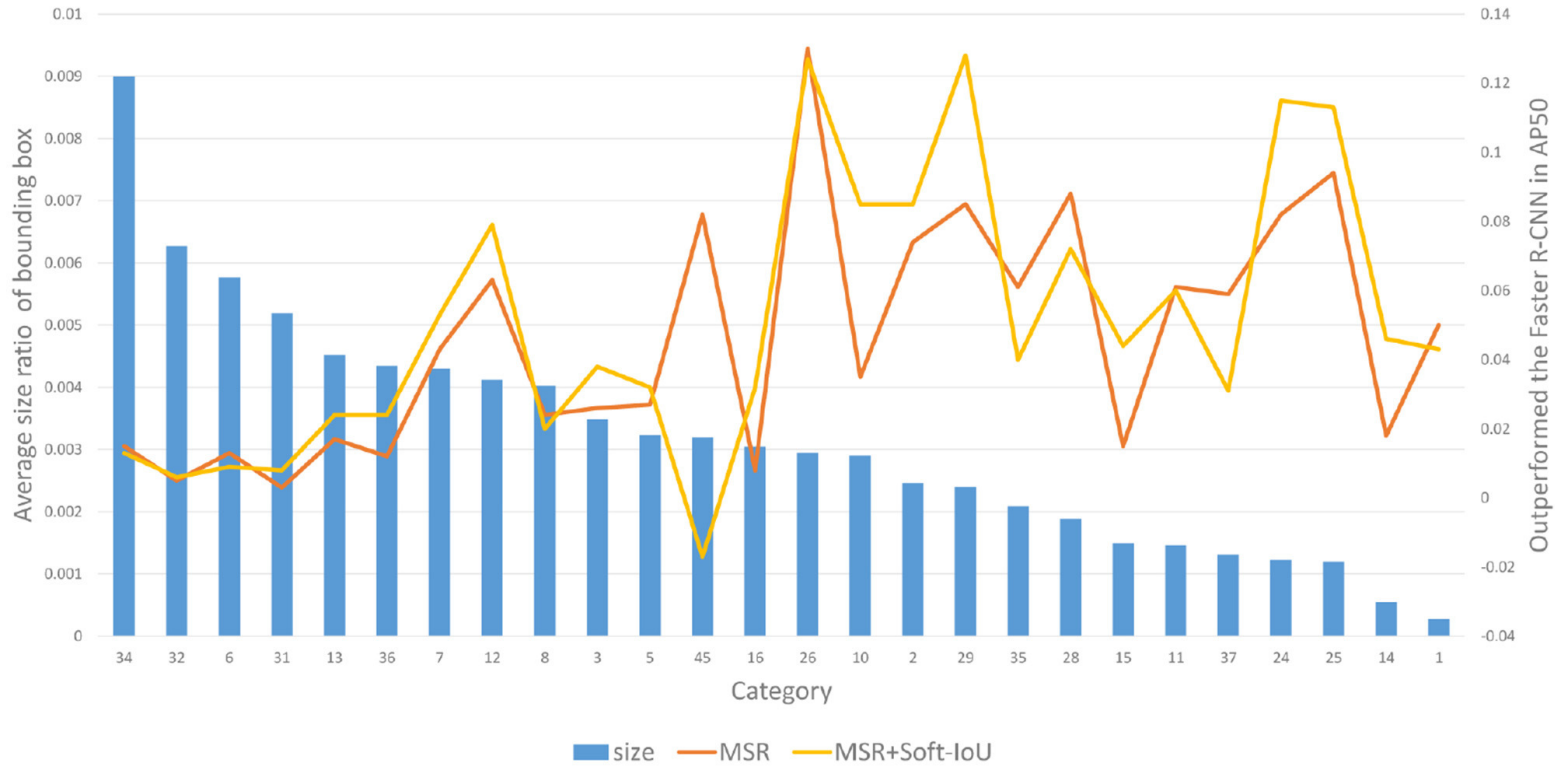
Improved performance of our MSR-RCNN on pest data of different sizes.

Figure 6 shows the performance improvement of our MSR-RCNN compared with Faster R-CNN. Among them, the blue bar chart represents the size of the pest, and the line chart describes the performance improvement of the method for Faster R-CNN. Our methods (MSR and SI) mainly improve the detection performance of small-size objects. For medium-size pests, the performance of Soft-IoU is improved significantly.

##### 4.2.2.2. The Training Loss and AP

**Figure 7:**
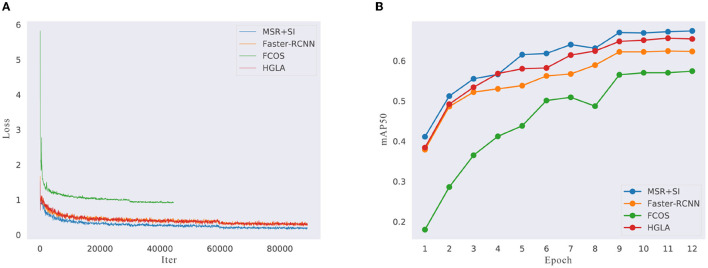
The training loss and mAP_50_. **(A)** The comparison of training loss. **(B)** The comparison of test accuracy.

To explain the improvement of our network in more detail, we present the training loss diagram of MSR-RCNN, Faster R-CNN, FCOS, and HGLA, as shown in Figure 7. Faster R-CNN represents two-stage methods, FCOS represents one-stage methods, and HGLA represents pest detection methods. Referring to the parameter setting of MMdetection, the batch size of FCOS is 4 samples, thus the loss iter only has half the other methods. It is clear that compared with other networks, our MSR-RCNN has more excellent data fitting ability and is capable of more complex work. In addition, our MSR-RCNN convergence rate is the fastest.

##### 4.2.2.3. The Beta Value

**Figure 8:**
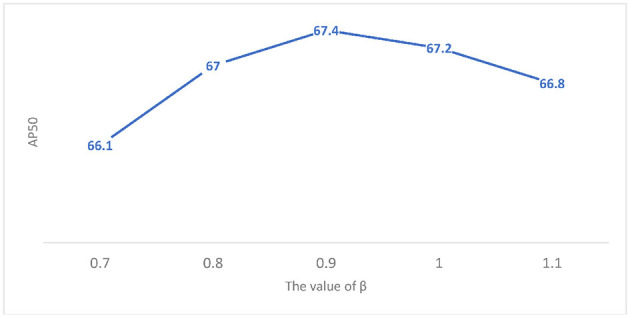
Ablation of β in the Soft-IoU (SI).

For the β in Formula (4), an ablation study is performed and the results are shown in Figure 8. When the β is less than 0.9, the detector performance is affected because a large number of positive samples change into negative samples, resulting in the imbalance between positive and negative samples. When the β is greater than 0.9, the training performance of the model is misled due to the addition of too many low-quality detection boxes.

##### 4.2.2.4. The Backbone of Our MSR Pest Detection Network

We choose ResNet50 as the backbone of the MSR-RCNN After a detailed comparison of the common backbone network. Table 3 shows the performance comparison of our MSR-RCNN in different backbone networks. Why the result of ResNet50 is better than ResNet101? This reason is that the object size is generally small in our dataset. Therefore, with the deepening of the network layer, the features of small-size objects gradually disappear in the continuous convolution operation. The top-to-down feature fusion method transmits blurry semantic information resulting in decreasing performance. To be fair, ResNet50 is used as the backbone extraction network for all comparative experiments in this study, unless otherwise stated.

**Table 3:** MSR-RCNN network performance comparison results using different backbones.

	**Resnet50**	**Resnet101**	**Resnext50**	**Resnext101**
*AP*_50_	**66.9**	66.1	66.3	66.7
*AP*_75_	40.0	39.4	**40.3**	39.6
*AP*	**38.0**	37.4	**38.0**	37.8

*The parts in bold represent the best performance*.

##### 4.2.2.5. MSR Module With Various Networks

We compare the performance of our MSR module with Faster R-CNN, Cascade R-CNN, FCOS, and RetinaNet, as shown in Table 4. The Faster R-CNN use the C4 feature map to detect pest. Due to the design of FPN (Lin et al., 2017a), all methods after 2017 use the multi-layer features detection pattern. Without bells and whistles, the MSR module effectively improves the pest detection performance under various networks. The experimental results show that the MSR module can improve the feature extraction capability and replace FPN in the pest detection field.

**Table 4:** The performance of MSR with various detection methods.

**Method**	**MSR**	***AP***	***AP*_50_**	***AP*_75_**	***mRecall***
Faster R-CNN (Ren et al., 2017)		34.8	61.8	36.1	51.5
Faster R-CNN + FPN (Lin et al., 2017a)		35.4	62.3	37.7	50.5
Faster R-CNN + MSR	√	**37.6**	**66.3**	**39.5**	**51.9**
Cascade R-CNN + FPN (Cai and Vasconcelos, 2018)		36.0	62.6	38.5	50.2
Cascade R-CNN + MSR	√	**37.8**	**65.8**	**40.2**	**52.1**
FCOS + FPN (Tian et al., 2019)		33.1	57.0	35.9	**55.3**
FCOS + MSR	√	**33.8**	**58.8**	**36.0**	54.8
RetinaNet + FPN (Lin et al., 2017b)		27.9	48.8	29.4	**53.1**
RetinaNet + MSR	√	**30.8**	**53.1**	**33.3**	52.7

*The parts in bold represent the best performance*.

#### 4.2.3. Generalization Capacity

We compare the performance on general object detection datasets (PASCAL VOC and COCO), as shown in Table 5. Where * represents the results that we reproduced with MMDetection under the same parameter settings. Due to the Soft-IoU being designed for pest detection, we only present the performance of MSR-RCNN with the MSR module. Since MSR-RCNN is a small-size detection network for pest detection, we do not evaluate the performance of *AP*_*l*_. The training set of PASCAL VOC 0712 is used to train networks and the test set of PASCAL VOC 2007 is used to verify the results. The experimental results show that our method can significantly improve the performance of *IoU*_50_ and small-size objects. This is highly consistent with the original intention of our MSR module.

**Table 5:** Detection performance comparison on general object detection datasets.

**Benchmark**	**Method**	**Backbone**	***AP***	***AP*_50_**	***AP*_75_**	***AP*_*s*_**	***AP*_*m*_**
PASCAL VOC	Faster R-CNN^*^	Resnet50	-	81.0	-	-	-
	MSR-RCNN		-	81.8	-	-	-
COCO	Faster R-CNN^*^	Resnet50	37.4	58.1	40.4	21.2	41.0
	MSR-RCNN		37.5	59.8	40.0	21.7	41.4

*Where*

* * represents the method of reproduction using MMdetection*.

**Figure 9:**
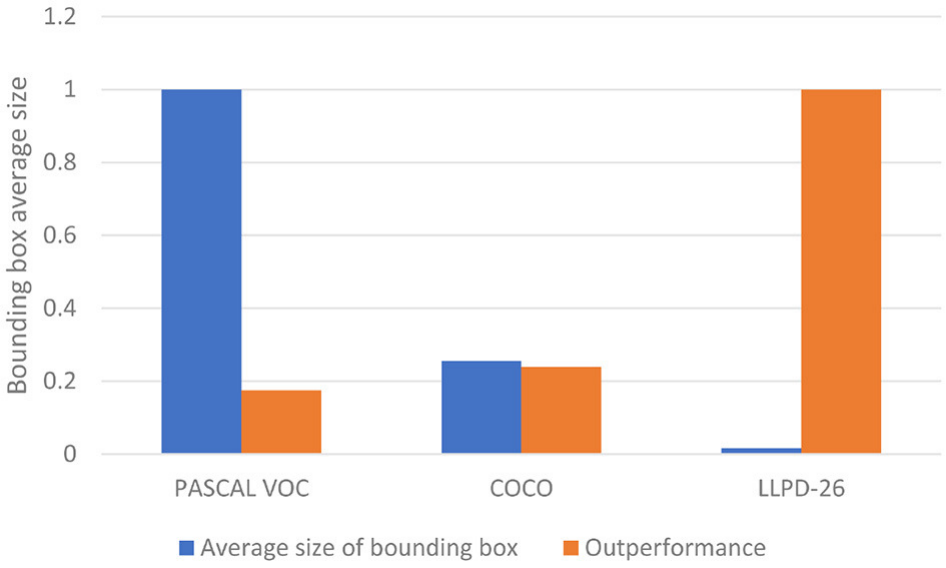
The performance comparison between MSR-RCNN and Faster R-CNN on different datasets.

In addition, Figure 9 shows the performance comparison between our method and Faster R-CNN on different datasets, where the blue bar chart represents the normalized relative average size of the objects in several datasets, the yellow bar chart shows the normalized relative AP improved by our MSR-RCNN method compared to Faster R-CNN. With the increase of the object average size, the improvement of the performance becomes more and more obvious.

### 4.3. Qualitative Results

**Figure 10:**
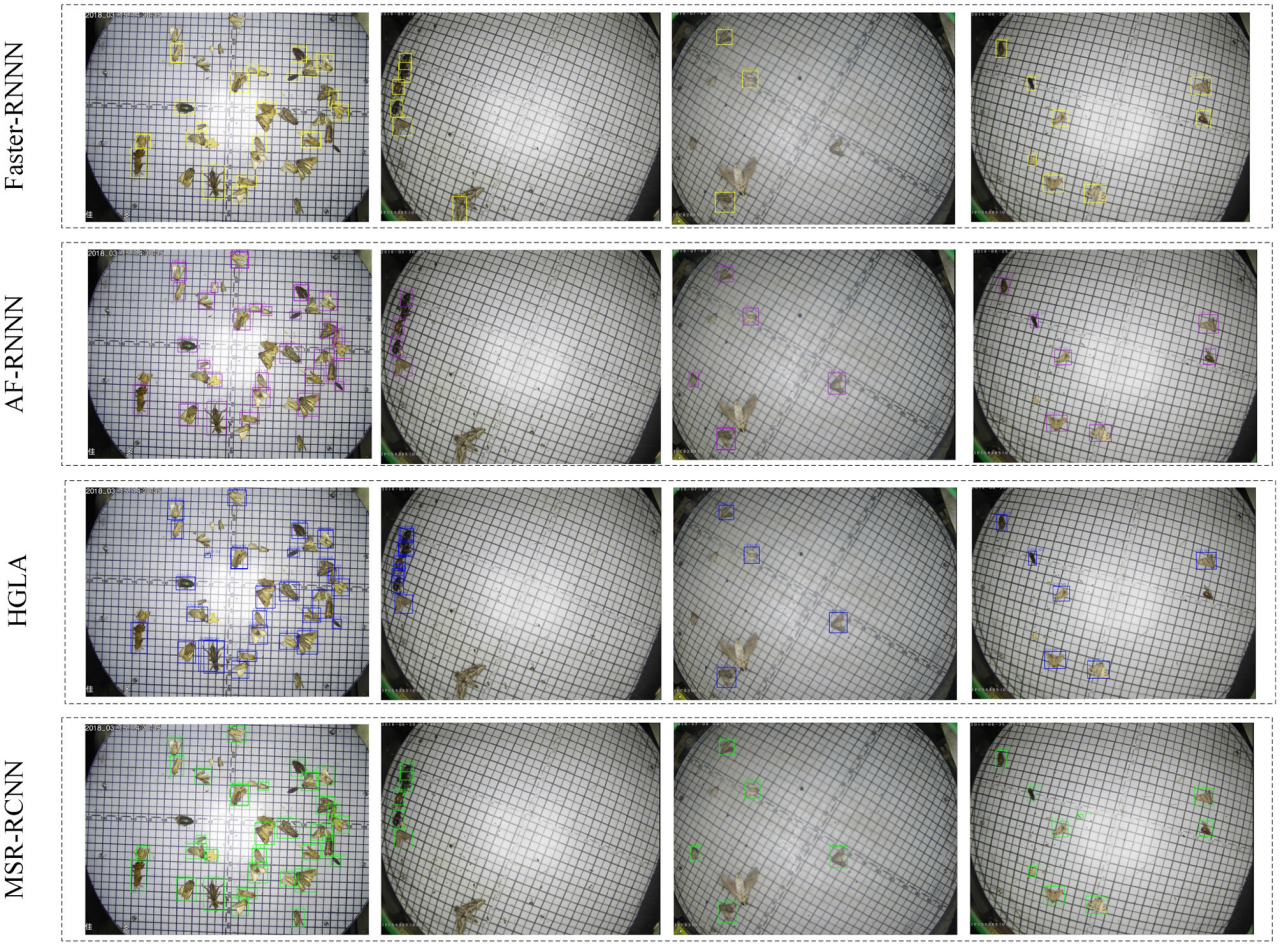
Visualization results.

To visually observe the accuracy, we visualize the detection results of Faster R-CNN, AF-RCNN, HGLA, and MSR-RCNN (ours), as shown in Figure 10. Among them, the first column shows the dense distribution pest images, the second and fourth columns show the sparse distribution pest images, and the third column shows the image detection results when the camera has water mist caused by temperature change. The visualization shows that HGLA has many overlapped bounding boxes, AF-RCNN and Faster R-CNN mainly exhibit missed bounding boxes and false results (Figure 10 columns 1 and 2). For columns 3 in Figure 10 (low-quality images caused by equipment reasons), all of the detection results are degraded, but our MSR-RCNN is the least weakened. This is owed to our feature super-resolution module. Although the MSR-RCNN wrongly identifies the rice planthopper in the fourth column images (class 1 is identified as class 14), other methods did not find the existence of minimum-sized pests (Figure 10 columns 4). The visualization results show that our MSR-RCNN outperforms other methods.

## 5. Conclusion

This study aims to bridge the gap between generic object detection and pest detection, in which the challenges lie in object characters and IoU adaptation. Therefore, we propose an MSR-RCNN that is targeted at detecting agricultural pests of 26 categories. Specifically, we build a large-scale light-trap pest dataset LLPD-26. For tackling the detection difficulty on small-size, multi-scale, and high-similarly pests, the MSR-RCNN adopts a MSR model that includes a super-resolution component, a feature fusion mechanism, and a feature weighting mechanism. In addition, motivated by the higher importance of pest positions, we propose a SI strategy to improve the adaptability of the network. The experimental results show that the proposed method can effectively detect multiple classes of pests. Ablation experiments verify the MSR model can improve the performance of other detectors in the plug-and-play form. Future study will focus on few-shot pest detection research and real-world application deployment.

## Data Availability Statement

The original contributions presented in the study are included in the article/supplementary materials, further inquiries can be directed to the corresponding author/s.

## Author Contributions

YT contributed to the conception and design of software, analysis of the data and writing, and revising the manuscript. SD and SZ carried out compared method by using AF-RCNN and HGLA detector in experimental part. JZ and LL contributed to write and revise the manuscript. All authors contributed to the article and approved the submitted version.

## Funding

This study is supported in part by the national natural science foundation of China (no. 31671586) and the demonstration of intelligent management and control technology for the whole cycle of agricultural production (no. KFJ-STS-QYZD-167-02).

## Conflict of Interest

The authors declare that the research was conducted in the absence of any commercial or financial relationships that could be construed as a potential conflict of interest.

## Publisher's Note

All claims expressed in this article are solely those of the authors and do not necessarily represent those of their affiliated organizations, or those of the publisher, the editors and the reviewers. Any product that may be evaluated in this article, or claim that may be made by its manufacturer, is not guaranteed or endorsed by the publisher.
